# The Effect of Lithium on Inflammation-Associated Genes in Lipopolysaccharide-Activated Raw 264.7 Macrophages

**DOI:** 10.1155/2020/8340195

**Published:** 2020-07-25

**Authors:** Raymond T. Makola, Vusi G. Mbazima, Matlou P. Mokgotho, Vincent S. Gallicchio, Thabe M. Matsebatlela

**Affiliations:** ^1^Department of Biochemistry Microbiology and Biotechnology, School of Molecular and Life Sciences, University of Limpopo (Turfloop Campus), Sovenga 0727, South Africa; ^2^Department of Biological Sciences, Clemson University, Clemson, SC 29634, USA

## Abstract

Lithium remains the preferred Food and Drug Administration- (FDA-) approved psychiatric drug for treatment of bipolar disorders since its medical establishment more than half a century ago. Recent studies revealed a promising role for lithium in the regulation of inflammation, oxidative stress, and neurodegeneration *albeit* unclear about its exact mode of action. Thus, the intention of this study is to delineate the regulatory mechanisms of lithium on oxidative stress in lipopolysaccharide- (LPS-) activated macrophages by evaluating its effects on nuclear factor-*κ*B (NF-*κ*B) activity and mRNA expression of multiple oxidative stress-related NF-*κ*B genes. Raw 264.7 macrophages were treated with up to 10 mM lithium, and no change in cell proliferation, viability, growth, and cell adhesion was observed in real time. Pretreatment with low doses of lithium was shown to reduce nitric oxide (NO) production in LPS-activated macrophages. A reduced internal H_2_DCFDA fluorescence intensity, indicative of reduced reactive oxygen species (ROS) production, was observed in LPS-activated Raw 264.7 macrophages treated with lithium. Lithium has been shown to lower the production of the chemokine RANTES; furthermore, this inhibitory action of lithium has been suggested to be independent of glycogen synthase kinase-3 *β* (GSK3*β*) activity. It is shown here that lithium modulates the expression of several inflammatory genes including I*κ*B-*α*, TRAF3, Tollip, and NF-*κ*B1/p50 which are regulators of the NF-*κ*B pathway. Moreover, lithium inhibits NF-*κ*B activity by lowering nuclear translocation of NF-*κ*B in LPS-activated macrophages. This is the first study to associate Tollip, Traf-3, and I*κ*B-*α* mRNA expression with lithium effect on NF-*κ*B activity in LPS-activated Raw 264.7 macrophages. Although these effects were obtained using extratherapeutic concentrations of lithium, results of this study provide useful information towards understanding the mode of action of lithium. This study associates lithium with reduced oxidative stress in LPS-activated Raw 264.7 macrophages and further suggests candidate molecular targets for the regulation of oxidative stress-related diseases using lithium beyond bipolar disorders.

## 1. Introduction

Lithium is a gold standard therapeutic drug used in psychiatry for the treatment of manic depression since its establishment in 1949 [1, 2]. It is an alkali metal and a monovalent trace element known to be reactive due to its unpaired electron which contributes to its chemical reactivity [3]. Lithium regulates several major biological processes which include receptor-mediated signalling, ion transport, hormonal and circadian systems, and inflammatory signalling pathways [3]. Lithium exerts most of its effects on several bioprocesses through inhibition or activation of vital enzymes such as inhibition of inositol monophosphatase (IMPase), inositol polyphosphate 1-phosphatase (IPPase), bisphosphate 3-nucleotidase (BPNT1), fructose 1,6 bisphosphate (FBPase), nuclear factor-E2 factor 2 (Nrf 2), cyclooxygenase (COX), and GSK-3*β* [4]. This enzyme inhibition/activation phenomenon is thought to be the mechanism behind lithium alteration of cell signalling pathways [5].

Lithium is thought to inhibit or activate some of these enzymatic activities partly due to its similar radius with enzymes cofactors such as magnesium [6]. Interference of lithium with vital inflammatory kinases and transcription factors makes it a potential candidate for regulation of chronic inflammation and oxidative stress conditions. A larger group of chronic ailments are related to or caused by dysregulation of the innate immune response and uncontrolled production of reactive oxygen species that result in oxidative stress. Oxidative stress occurs when the production of oxidants outweighs the production of antioxidants such as superoxide dismutase, glutathione peroxidase, and catalase as well as the nonenzymatic antioxidants such as glutathione and vitamins C, D, and E. Under normal physiological conditions, production of antioxidants serves as a defence mechanism that neutralises detrimental free radicals such as reactive oxygen species (ROS) and reactive nitrogen species (RNS) [7].

These ROS are normal byproducts of metabolism generated by reducing oxygen during mitochondrial electron transport chain. They are known to be less severe in trace amounts and yield beneficial properties that include wound healing and cellular signalling pathways as second messengers [7]. Most ROS are produced in excess during inflammation, the oldest known defence mechanism both phylogenetically and ontogenetically. Inflammation occurs as an immune response to an external or internal challenge by an injurious agent. It is controlled by cytokines, chemokines, products of the plasma enzyme systems, lipid mediators released from different cells and vasoactive mediators released from mast cells, basophils, platelets, and macrophages [8].

Inflammation and oxidative stress conditions emanate from persistent activation of inflammatory enzymes and transcription factors such as activator protein -1 (AP-1), NF-*κ*B, and GSK-3*β* [9, 10]. In other studies, lithium has been postulated to inhibit GSK-3*β* both directly through competitive inhibition that includes binding to magnesium-sensitive sites and indirectly through induced phosphorylation at serine-9/21 residue by protein kinases C and B [2, 11]. Although the putative mechanism by which lithium exerts its antimanic, antidepressant, and antiapoptotic characteristics is not well known, other studies [4, 5] link these lithium properties with the inhibition of GSK-3. The enzyme GSK-3 is a serine-threonine kinase previously known for its role in insulin receptor signalling; however, recent findings demonstrated the broad spectrum of GSK-3 activities such as its involvement in cell growth, differentiation, apoptosis, and inflammation [12, 13].

GSK-3 is thought to regulate these bioprocesses through the modulation of several cell signalling pathways and activation of transcription factors and enzymes such as AP-1, cAMP response element-binding (CREB), NF-ĸB, heat shock protein 1 (HSP-1), and CCAAT/enhancer binding proteins [12, 13]. Lithium's antimanic, antidepressant, and anti-inflammatory properties mode of action remains sparse, since most of these reports emanate from neuropsychiatry experimental models [12–14]. Hence, in this study, oxidative stress related genes are investigated in the macrophage model as an attempt to further delineate its mode of action. In this study, the effects of lithium on cellular integrity and mRNA expression of genes known to play a role in inflammation and oxidative stress are investigated in LPS-activated Raw 264.7 macrophages in real time.

## 2. Results

### 2.1. Effects of Lithium on Morphology, Adhesion, Growth, and Viability of Raw 264.7 Cells

**Figure 1:**
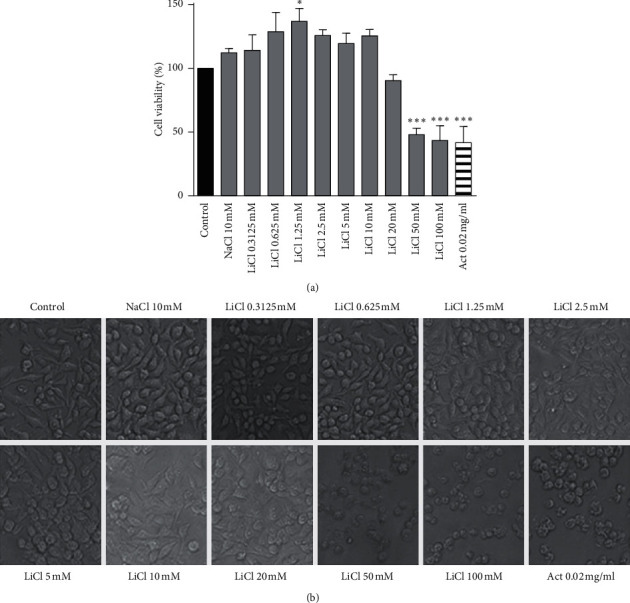
The effects of lithium on viability, morphology, and proliferation of Raw 264.7 macrophage cells. To examine the effects of lithium on proliferation and viability of Raw 264.7 macrophages, cells were seeded in 96-well plates at 6 × 10^6^ cells/well and treated with LiCl (0.3125, 0.625, 1.25, 2.5, 5, 10, 2, 0, 50, and 100 mM), NaCl (10 mM), and actinomycin-D (0.02 mg/ml) for 24 hrs. Cells were viewed and captured with the Nikon Ti-E inverted microscope (b); thereafter 5 mg/ml MTT was added for 4 hrs and then absorbance was read using by GloMax-Multi microplate reader at 570 nm (Promega, USA) (a). Graphs were executed using GraphPad Prism 4 software and the statistical analysis was carried out using GraphPad InStat™ software using ANOVA (Tukey–Kramer, ^*∗*^*p* < 0.05; ^*∗∗∗*^*p* < 0.001).

**Figure 2:**
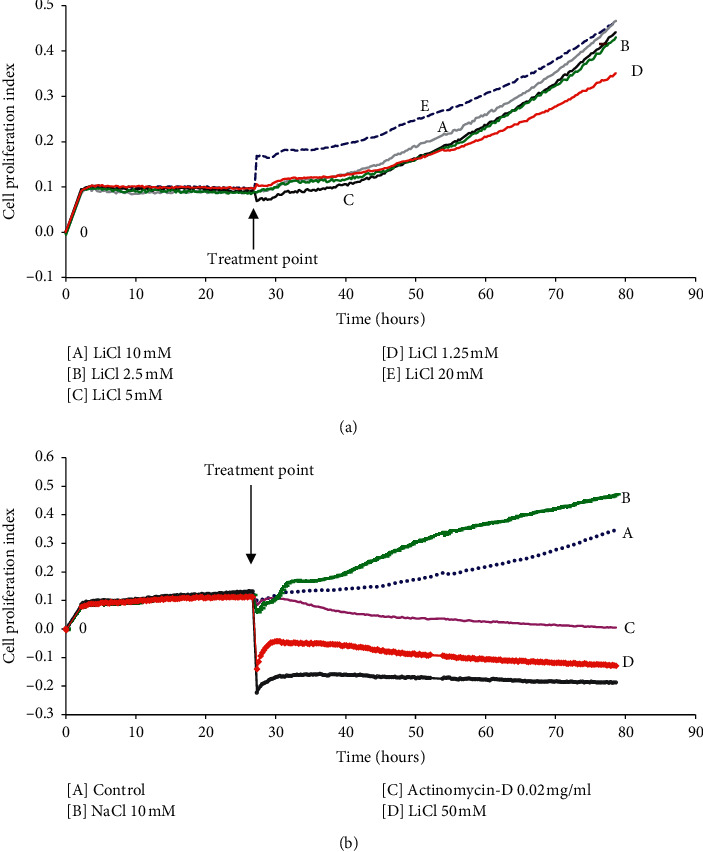
The effect of lithium on proliferation and cellular integrity of Raw 264.7 macrophages in real time. Cells were seeded at 40,000 cell/well in an E plate for 24 hrs and then treated with 0.02 mg/ml actinomycin-D, 10 mM NaCl, and various concentrations of lithium (1.25–20 mM (a) and 50–100 mM (b)) for 56 hrs. The xCELLigence (RTCA) software was set to take readings after every 15-minute interval giving a real-time measure of cell size, adhesion, growth, viability, and proliferation.

To evaluate the cytotoxicity profiles of lithium on Raw 264.7 macrophages, cells were treated with various lithium concentrations wherein cell viability and integrity was evaluated in real time using the MTT viability assay and xCELLigence real-time cell analyser system. Treatment of Raw 264.7 cells with 0.3125–20 mM lithium resulted in no change in cell viability as compared to negative control; 0.02 mg/ml actinomycin-D and 50–100 mM lithium which were highly cytotoxic (Figure 1(a)). Moreover, the Raw 264.7 cells retained their morphology after treatment with lithium. As depicted in the cell images below, LiCl concentrations between 0.3125 and 10 mM stimulated growth of these immune cells, which is not the case with 20 mM LiCl (Figure 1(b)). In addition, the current-based real-time cell analyser system (xCELLigence) showed that concentrations of up to 20 mM lithium did not induce any form of cell death in Raw 264.7 cells (Figure 2(a)), whereas 50–100 mM lithium concentrations were as cytotoxic as 0.02 mg/ml actinomycin-D (Figure 2(b)). Control cells, NIH 3T3 fibroblast cells, showed similar outcomes as treated with various concentrations of lithium (results not shown).

### 2.2. Effects of Lithium on Induction of Apoptosis in Raw 264.7 Macrophages

**Figure 3:**
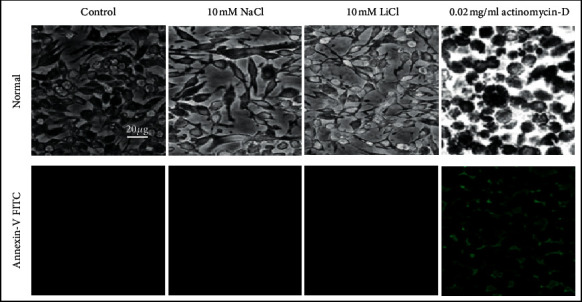
Determination of the apoptosis effect of lithium on Raw 264.7 macrophage cells using fluorescent microscopy. Cells were cultured at a density of 6 × 10 ^5^ cell/ml on coverslip in 6-well plates. Followed by treating cell with lithium 10 mM, NaCl 10 mM, actinomycin-D 0.02 mg/ml, and untreated cell were used as control for 24 hrs. Staining with Annexin-V and PI followed for 30 min at RT in the dark. Thereafter, cells were fixed and mounted on the slides and then measurement of fluorescence was accomplished by capturing pictures at 20x magnification with Nikon Ti-E inverted fluorescent microscope.

**Figure 4:**
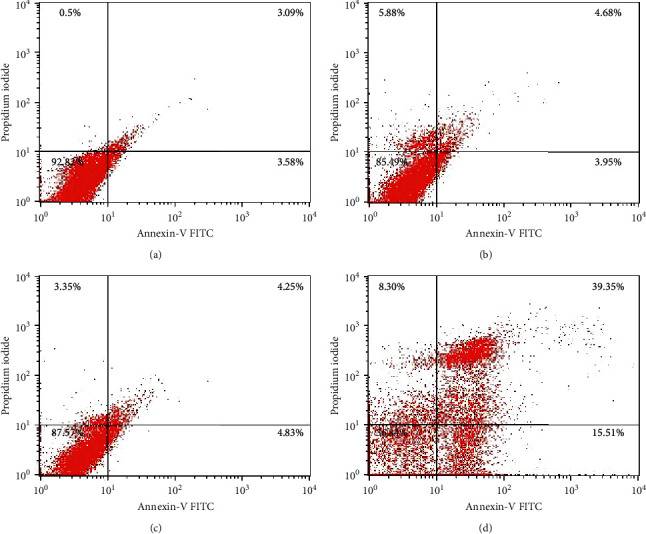
Determination of apoptotic properties shown by lithium on Raw 264.7 macrophage cells using flow cytometry. Apoptosis investigation was carried out by seeding cells at a concentration of 6 × 10^6^ cells/ml overnight in a 200 mm dish. This was then followed by treating the cells with 5, 10, 20, and 50 mM lithium, 10 mM NaCl, and 0.01 and 0.02 mg/ml actinomycin-D for 24 hrs. Cells were thereafter stained with mixture of binding buffer, Annexin-V FITC, and PI for 20 min in the dark, and then cell analysis was carried out using flow cytometer (BD Biosciences, USA) according to the manufacturer's protocol. (a) Untreated control. (b) LiCl 10 mM. (c) NaCl 10 mM. (d) Actinomycin-D 0.02 mg/ml.

To evaluate the induction of apoptosis in macrophages, cells were treated with 10 mM lithium for 24 hours and stained with Annexin-V-FITC and PI. Cells staining positive for Annexin-V-FITC are indicative of the presence of phosphatidylserine (PS) residues on membrane surface and early apoptosis, whereas nuclei staining positive with PI indicate loss of membrane integrity and late apoptosis. Raw 264.7 cells treated with 10 mM lithium retained their morphology and stained negative for both Annexin-V and PI (Figure 3). On the other hand, cells treated with 0.02 mg/ml actinomycin-D lost their spindled shape morphology and stained positive for Annexin-V but negative for PI (Figure 3). In addition to microscopic analysis of apoptotic effects of lithium, flow cytometric analysis further showed the nonapoptotic property of 10 mM lithium (Figure 4). Untreated control, 10 mM NaCl-, and 10 mM lithium-treated cells maintained a high cell count in the negative bottom-left quadrat as shown by quadrat statistics (92.82%, 87.57%, and 85.49%, resp.). The negative control 0.02 mg/ml actinomycin-D treatment displayed a major cell count in the upper-right (39.35%) and a minor cell count in the bottom-right quadrat (15.51%), thus, suggesting the onset of the late phase of apoptosis.

### 2.3. Lithium Inhibits Oxidative Burst in LPS-Activated Raw 264.7 Cells

**Figure 5:**
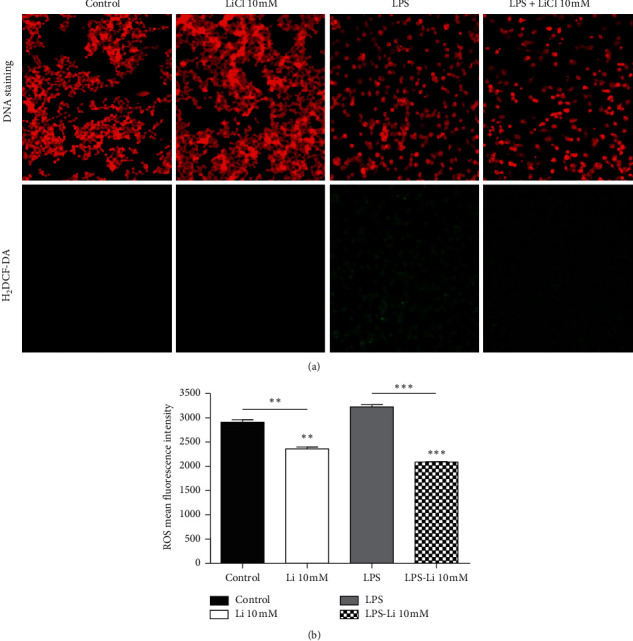
Effect of lithium on oxidative burst activated by LPS on Raw 264.7 macrophage cells. Cells were seeded at a density of 6 × 10^5^ cells/ml (200 *μ*l) on a coverslip and pretreated with 10 mM lithium and treated with 10 *μ*g/ml LPS for 24 hrs. The fluorescence measurement was accomplished with inverted fluorescent microscope Nikon Ti-E at 10x magnification (a). Fluorescence intensity of the pictures was measured using the NIS Element view imaging software at 10x magnification (Nikon, Japan). Graphs were executed using GraphPad Prism 4 software and the statistical analysis was carried with GraphPad InStat software using ANOVA (Tukey–Kramer) (^*∗*^*p* < 0.05; ^*∗∗∗*^*p* < 0.001).

The key role played by macrophages as the first line of defence and that they express important receptors that stimulate inflammatory signalling pathways makes this cell line a priority in this study [15]. The persistent/undercontrolled stimulated inflammation elicits oxidative stress which is linked to the pathogenesis of a plethora chronic ailments [7], hence the use of stimulated Raw 264.7 as the model for oxidative stress. As displayed in Figure 5(a), the lower fluorescence intensity observed in Raw 264.7 cells after treatment with 10 mM lithium suggests that lithium inhibits production of ROS in these cells. The LPS-activated Raw 264.7 cells displayed high fluorescence intensity compared to cells treated with both LPS and 10 mM lithium. The variation in fluorescence intensity was as well shown by the measurement of fluorescence intensity using the NIS Element view imaging software (Nikon, Japan) (Figures 5(a) and 5(b)). These findings show a statistically significant difference of fluorescence intensity between cells treated with lithium alone and those treated with both lithium and LPS (Figure 5(b)). Other stimulants such as FMLP and PMA have been used in this study. The treatment of cells with lithium in the presence of those stimulants showed lowered fluorescence intensity (results are not shown).

### 2.4. Effects of Lithium on LPS-Induced NO Production on Raw 264.7 Cells

**Figure 6:**
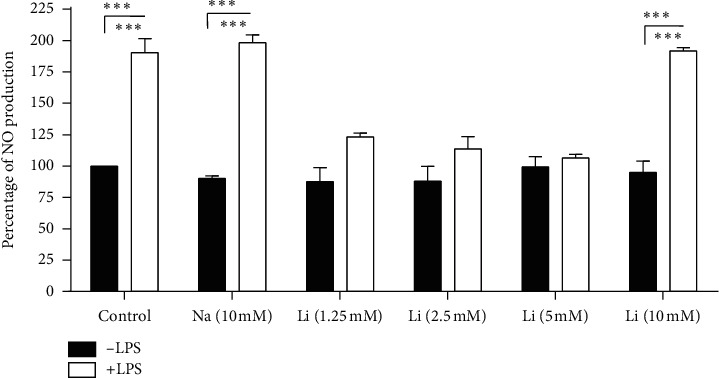
Effect of lithium on nitric oxide (NO) production assessed using Griess reagent on LPS-activated Raw 264.7 cells. Cells were seeded at a density of 6 × 10^6^ cells/ml and treatment was executed by pretreating with 10 mM NaCl and lithium (1.25, 2.5, 5, and 10 mM) for an hour thereafter; other set of wells that are already treated with lithium and NaCl were stimulated with 10 *μ*g/ml LPS for 24 hrs. Quantitation of NO production was carried out by GloMax-Multi microplate at 550 nm (Promega, USA). Treatments that encompass various concentrations of lithium and 10 mM NaCl combined with LPS were compared with LPS activated wells. Graphs were executed using GraphPad Prism 4 software and the statistical analysis was carried out using GraphPad InStat software and ANOVA (Tukey–Kramer) (^*∗*^*p* < 0.05; ^*∗∗∗*^*p* < 0.001).

**Figure 7:**
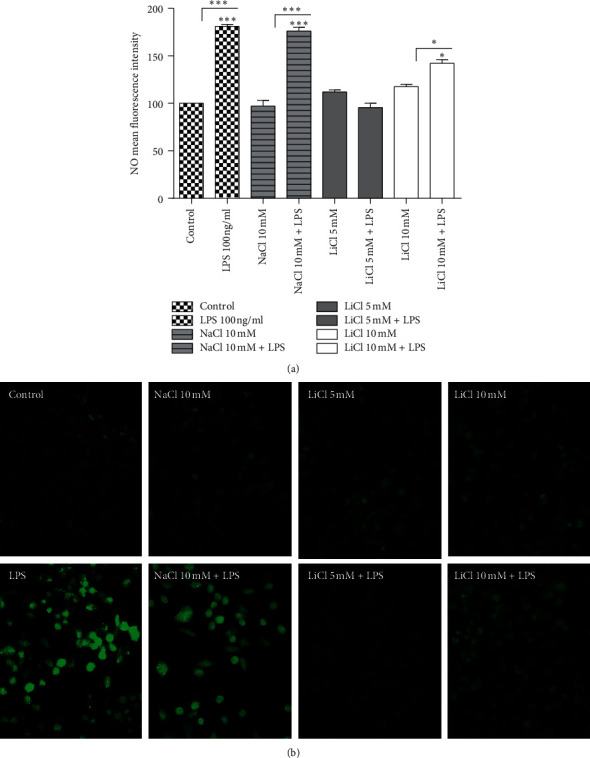
Determination of effect of lithium on nitric oxide production on LPS-induced Raw 264.7 cells. Cells were seeded at 6 × 10^6^ cell/ml and treated with lithium (5 and 10 mM), 10 mM NaCl, and 100 ng/ml LPS, combining each of the chemicals with LPS for 24 hrs. Cells were stained with DAF2-DA and Hoechst for 20 min at RT. The measurement of the fluorescence was accomplished by using fluorescent inverted Nikon Ti-E microscope at 20x magnification (b). Fluorescence intensity of the pictures was measured using the NIS Element view imaging software (Nikon, Japan) (a). Graphs were executed using GraphPad Prism 4 software and the statistical analysis was carried out using GraphPad InStat software and ANOVA (Tukey–Kramer) (^*∗*^*p* < 0.05; ^*∗∗∗*^*p* < 0.001).

Activated macrophages produce nitric oxide which has a short life-span due to its instability and volatility. Analysis of nitric oxide involves colorimetric measurement of nitrite (NO_2_‐), its stable and nonvolatile breakdown product [16]. Nitric oxide measure embodies levels of reactive nitrogen species (RNS). Lithium- (1.25, 2.5, and 5 mM) treated cells significantly inhibited NO production in LPS-activated Raw 264.7 cells compared to untreated LPS- activated control cells. However, the 10 mM lithium-treated cells and LPS-activated cells showed no inhibition of NO production. The same outcomes were observed in stimulated 10 mM NaCl-treated cells (*p* < 0.05) (Figure 6). The findings in Figure 6 were also confirmed using a nitric oxide fluorescent probe, DAF2-DA, which showed that less than 10 mM lithium concentrations significantly lower NO production. Low fluorescence intensity was detected in cells treated with less than 10 mM doses of lithium and activated with LPS compared to cells activated with LPS alone. A lower fluorescence intensity was observed in LPS-activated macrophages treated with 5 mM lithium compared to LPS-activated untreated control and NaCl-treated cells (Figures 7(a) and 7(b)).

### 2.5. The Effects of Lithium on Translocation of the NF-*κ*B Transcription Factor in Raw 264.7 Macrophages Activated with LPS

Since NF-*κ*B is one of the main transcription factors involved in the regulation of inflammation and ROS/RNS production, the effect of lithium on translocation of this transcription factor in LPS-activated macrophages was studied. Upon activation, the NF-*κ*B dimer (p50/p65) translocates from the cytoplasm into the nucleus where it binds to kappa responsive site and induce transcription of several inflammatory genes. Hence, translocation of NF-*κ*B from cytosol to nucleus is indicative of onset of inflammation. Thus, LPS-activated Raw 264.6 macrophages were treated with lithium, and NF-*κ*B translocation was monitored using immunofluorescence microscopy. The position of the nuclei is marked with DAPI-staining, and the movement of the NF-*κ*B dimer is monitored using antibodies against NF-*κ*B (p65) with secondary antibodies tagged with a green fluorescent probe (FITC).

**Figure 8:**
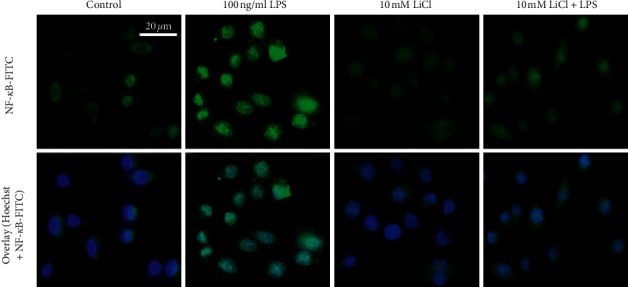
Evaluation of the effects of lithium on nuclear translocation of NF-*κ*B after LPS activation of Raw 264.7 cells. Cells were cultured at a density of 2 × 10^5^ cells/ml on coverslips and treated with 10 mM lithium, 10 mM NaCl, 100 ng/ml ultrapure LPS, and a combination of salts and LPS for 90 min. Cells were then fixed with 3.7% paraformaldehyde and thereafter semipermeabilised with 0.1% Triton-X100 in 1% BSA for 30 min. The nonspecific binding was blocked for an hour with 1% BSA and then anti-NF-*κ*B antibody (1 : 500) was added for an hour, and then the cells were washed 3x with 1x PBS and stained for 30 min with anti-rabbit IgG-FITC (1 : 2000). After 30 min incubation, cells were washed, followed by DNA staining with 25 *μ*g/ml Hoechst for 20 min. Thereafter, coverslips were mounted on glass slides with mounting medium and then NF-kB was analysed and photographed under fluorescence inverted microscope (Nikon Eclipse TS100F, Japan).

A high amount of green fluorescence was observed in the nuclei of LPS-activated macrophages showing increased presence of the NF-*κ*B dimer in the nucleus (Figure 8). Treatment of these activated macrophages with lithium lowered the presence of fluorescent NF-*κ*B in the nuclei. The results below show that lithium inhibits the translocation of the NF-*κ*B from the cytoplasm to the nucleus in LPS-activated Raw 264.7 macrophages. In untreated macrophages, the NF-*κ*B dimer is located in the cytosol as shown by a characteristic green fluorescence within the cytoplasm and a conspicuous absence of the dimer in the nuclei as overlaid with DAPI-stained nucleus position (Figure 8).

### 2.6. Lithium Modulates Overexpression of Inhibitory Molecules That Are Related to NF-*κ*B Signalling Raw 264.7 Cells after Being LPS Activated

**Figure 9:**
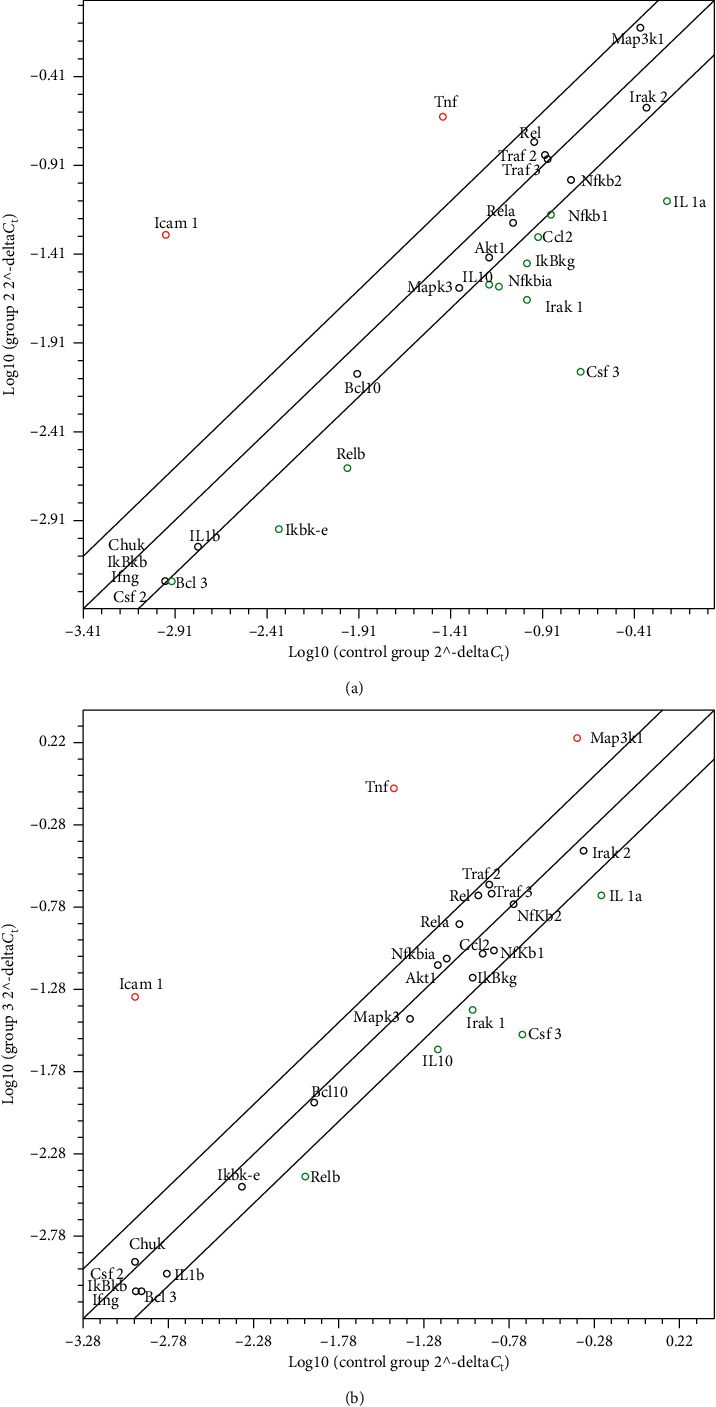
The expression pattern of NF-*κ*B signalling-related genes on Raw 264.7 cells activated with LPS. Cells were cultured in 60 mm cell culture dishes overnight at a density of 6 × 10^6^ cell/ml. Thereafter, cells were treated with 10 mM lithium, 10 *μ*m/ml LPS, and a combination of lithium with LPS for 24 hrs. Total RNA isolation was accomplished with Qiagen Total RNA isolation kit; cDNA was synthesised with RT^2^ first-strand kit. RT^2^ profiler PCR arrays were used to measure the expression profiles of NF-*κ*B in which 40 cycles where accomplished, 1 cycle for 10 min at 95°C and 40 cycles of 15 s at 95°C and 1 min at 60°C. Thereafter, Qiagen web-based data analysis was used to analyse the *C*_t_ values and generate the dot plots. (a) LPS vs. control. (b) Li-LPS vs. control.

**Figure 10:**
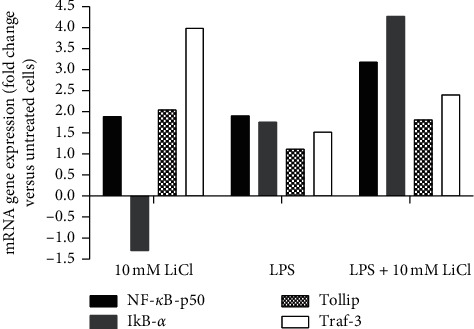
The effects of lithium on expression of NF-*κ*B signalling-related genes on Raw 264.7 cells activated with LPS. Cells were cultured in 60 mm cell culture dishes overnight at a density of 6 × 10^6^ cell/ml. Thereafter, cells were treated with 10 mM lithium, 10 *μ*m/ml LPS, and a combination of lithium and LPS for 24 hrs. Total RNA isolation was accomplished with Qiagen Total RNA isolation kit; cDNA was synthesised with RT^2^ first-strand kit. RT^2^ profiler PCR arrays were used to measure the expression profiles of NF-*κ*B in which 40 cycles were accomplished, 1 cycle for 10 min at 95°C and 40 cycles of 15 s at 95°C and 1 min at 60°C. Thereafter, Qiagen web-based data analysis was used to analyse the *C*_t_ values and then GraphPad Prism 4 was used to plot the graphs.

The real-time PCR array was used to determine the effect of lithium on mRNA expression levels of NF-*κ*B signalling-related genes in LPS-activated Raw 264.7 macrophages. In this experiment, changes in gene expression patterns were evaluated on NF-*κ*B signalling-associated genes such as NF-ΚB1/p50, Tollip, Traf-3, and I*κ*B-*α*. Treatment of nonactivated macrophages increased mRNA expression of NF-*κ*B1/p50, Tollip, and Traf-3 genes compared to the untreated control. Lithium increased macrophage NF-*κ*B1/p50 mRNA gene expression by 1.9-fold, Tollip levels were increased 2-fold, and a 4-fold increase in Traf-3 mRNA level was observed as compared to the untreated controls. Although a 4.5-fold increase in I*κ*B-*α* expression was observed in LPS-activated Raw 264.7 macrophage cells treated with 10 mM lithium, a 1.5-fold decrease in I*κ*B-*α* mRNA levels was observed in nonactivated macrophages treated with the same concentration of lithium (Figures 9 and 10). In LPS-activated macrophages, mRNA expression of NF-*κ*B1/p50, Tollip, Traf-3, and I*κ*B-*α* increased by 1.9, 1.0, 1.5, and 1.8-fold, respectively. When LPS-activated macrophages were treated with lithium, mRNA expression of NF-*κ*B1/p50 increased more than 3-fold, Tollip levels increased by 1.8-fold, Traf-3 levels increased by 2.4-fold, and I*κ*B-*α* mRNA levels increased by more than 4-fold compared to the untreated macrophages (Figures 9 and 10).

### 2.7. Lithium Inhibition Effect on the Production of Inflammatory Mediators Is Independent of GSK3*β*

**Figure 11:**
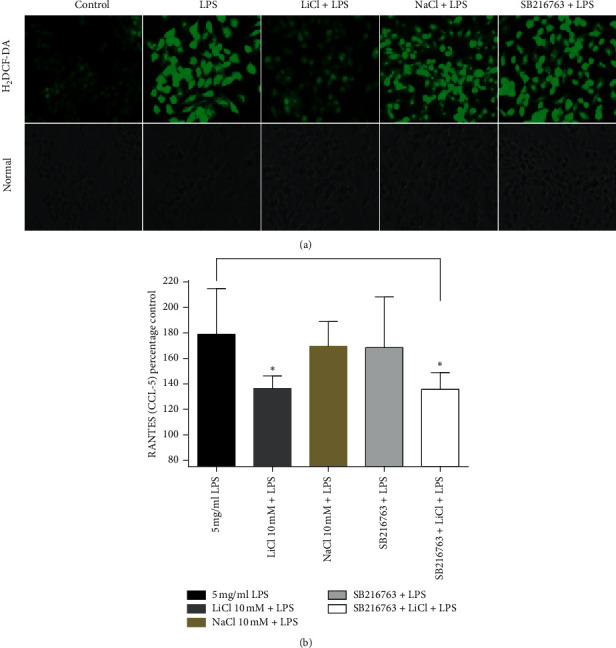
Effects of GSK3*β* inhibitors (lithium and SB216763) as mechanism of inflammation inhibition. Cells were seeded at 6 × 10^5^ cells/ml (200 *μ*l) on a coverslip and pretreated with 10 mM lithium/sodium chloride and activated with 5 mg/ml LPS for 24 hrs. The fluorescence measurement was captured at 20x magnification with EVOS FL Colour imaging system (Life technologies, USA). B measurement of the CCL-5 chemokine was executed by seeding cell at 6 × 10^6^ cells/ml in T25 flask overnight, followed by treatment of cells with lithium as outlined above and activated with 5 mg/ml LPS for 24 hrs. The levels of RANTES/CCL-5 in the supernatant were measured with Elisa assay 24 hrs after treatment and stimulation. The graphs were executed using GraphPad Prism 4 software and the statistical analysis was carried out with GraphPad InStat software using ANOVA (Tukey–Kramer) (^*∗*^*p* < 0.05; ^*∗∗∗*^*p* < 0.001).

Previous work links GSK3 to the regulation of the production of inflammatory mediators [9]. Thus, ROS and CCL-5 production assays (Figures 11(a) and 11(b)) were aimed at determining if lithium inhibits the production of these inflammatory molecules through GSK3 or through the inhibition of other enzymes. The SB216763 compound has been widely demonstrated to inhibit GSK-3*β* activity in both *in vitro* and *in vivo* models [17, 18]. In Raw 264.7 macrophage cells, 10 *μ*M SB216763 was previously shown to inhibit GSK-3*β* activity [19]. H_2_DCF-DA serves as a probe used to detect levels of ROS production, and a low fluorescence intensity was observed in cells treated with 10 mM LiCl and activated with 5 mg/ml LPS compared to the untreated 5 mg/ml LPS-activated cells. This inhibition phenomenon was not observed in cells that were treated with 10 *μ*M SB216763 and activated with 5 mg/ml LPS. Cells treated with 10 mM NaCl and 5 mg/ml LPS did not display any lowering of the fluorescence intensity (Figures 11(a)). The CCL-5 Elisa assay showed similar findings. Cells treated with lithium displayed lowered production of the CCL-5/RANTES chemokine (Figures 11(b)). There was no inhibition in the production of CCL-5 in cells treated with 10 µM SB216763 and activated with 5 mg/ml LPS. Moreover, 10 *μ*M SB216763 did not show abrogation of inhibitory properties of lithium on the production of CCL-5, since combination of lithium and SB216763 did not show any changes in inhibition of CCL-5. A combination of results from these experiments suggests that inhibition of inflammatory mediators by lithium may be independent of GSK3*β*.

## 3. Discussion

Pathogen recognition receptors (PRR) such as Toll-like receptors (TLRs) and interleukin-1 receptor type I (IL-1RI) expressed by the macrophages are central to mediating inflammatory processes. Macrophages play a crucial role as the first line of defence and create a link between innate and adaptive immune responses [15, 20]. Undercontrolled inflammatory response has been suggested to contribute to the pathogenesis of a number of ailments [7]; thus this work focused on the TLR-4-mediated NF-*κ*B signalling pathway. This is the first study to show that lithium regulates mRNA expression of I*κ*B-*α*, TRAF3, Tollip, and NF-*κ*B1/p50 while inhibiting oxidative stress in LPS-activated Raw 264.7 macrophages. The present study shows that lithium inhibits NO production, suppresses oxidative burst, and blocks NF-*κ*B nuclear translocation, thereby maintaining cell integrity and viability at doses not higher than 20 mM.

Using fluorescence-based cellular assays, lithium is shown to inhibit production of ROS and RNS in LPS-activated macrophages. Cell-based photographic images, flow cytometry, and real-time-based cellular assays are used to effectively present visual evidence that lithium exerts its anti-inflammatory effects in these activated macrophages without inducing detrimental morphological pressure or cell death. Although concentrations of lithium above 20 mM were used in various *in vitro* models [21, 22], this study has considered limiting concentrations of LiCl to 10 mM, since this has been demonstrated (Figure 1(a)) to be the highest lithium concentration with no effect on Raw 264.7 cell proliferation [20]. Although previous studies have suggested the narrow spectrum of action/activity shown by lithium at therapeutic concentrations (0.4–2.0 mM LiCl) [2, 23], this work, along with other studies performed at extratherapeutic dosages [24–26], explored the effects of lithium above those concentration limits so as to provide substantive molecular data on inflammation beyond the narrow spectrum.

**Figure 12:**
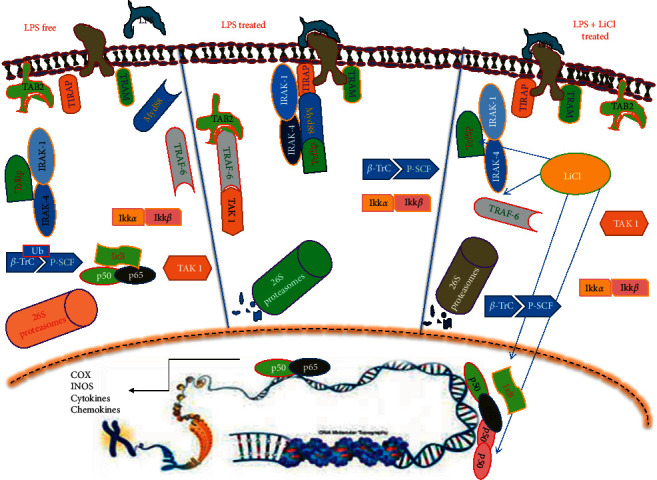
Demonstration of the putative mechanism of action of lithium in the NF-*κ*B signalling pathway. The LPS free signalling involves the inhibition of the IRAK proteins in the cytoplasm by Tollip protein; this molecule is known to arrest immune cells in quiescent state in the absence of infection or stimulant. Thus, the inhibition of the IRAK proteins leads to the integrity of I*κ*B; then the entire signalling pathway is inhibited. In the LPS treatment, the adapter molecule (Myd88) is recruited to the receptor; this then recruits the IRAK proteins in association with Tollip protein. Therefore, the IRAK protein phosphorylates TRAF protein which recruits the TAB2 and TAK 1 which phosphorylates activated IKK. The IKK then phosphorylates I*κ*B; this molecule is then tagged for degradation by a ubiquitin. This sequence sequesters the NF-*κ*B in the nucleus leading to the expression of inflammatory genes. The combinatory treatment that includes lithium chloride and LPS is thought to disturb the signalling pathway since lithium showed expression of some inhibitory proteins such as p50, I*κ*B-*α*, NF-*κ*B, and Tollip (shown by arrows). These inhibitory molecules are the control points of the NF-*κ*B signalling pathway. Overexpression of Tollip protein is thought to disturb the pathway by arresting the IRAK protein and then I*κ*B-*α* removes the NF-*κ*B from its responsive element by its DNA dissociation ability. Moreover, the p50 homodimer competes for the responsive element with the NF-*κ*B p50-p65 heterodimer.

This work presents NF-*κ*B as the main ROS and RNS regulator whose activity is inhibited by lithium possibly through gene modulation of its associate NF-*κ*B inhibitors: I*κ*B-*α*, TRAF3, Tollip, and NF-*κ*B1/p50.  Figure 12 stipulates the sequence of events in NF-*κ*B signalling pathway and lithium targeted proteins. Overexpression of Tollip impairs IL-1RI-, TLR2-, and TLR4-triggered NF-*κ*B and JNK signalling pathways. Tollip binds the activated IL-1RI complex, as well as TLR2 and TLR4 complexes. Tollip also interacts with IL-1R-associated kinase 1 (IRAK-1) prior to stimulation and suppresses IRAK-1 kinase activity [27]. This has led to the concept that a Tollip-mediated pathway is required to maintain immune cells in a quiescent state in the absence of infection and facilitate the termination of IL-1R- and TLR-induced signalling during inflammation via suppression of IRAK-1 activity [24, 27].

The onset of inflammation in LPS-activated macrophages is initiated by binding of LPS to TLR4 [28]. Thus, lithium could be inhibiting inflammation in LPS-activated macrophages by upregulating the expression of Tollip which would then suppress TLR4-triggered NF-*κ*B and JNK signalling pathways. Wang and colleagues have shown that inhibitory properties of lithium on IFN-*β* production in TLR3/4 and RIG-I-receptors mediated signalling which is suggested to involve the inhibition of TBK1, which could be the upstream inhibition of IRAK-1 by Tollip [20]. Moreover, this study suggests that the inflammatory influence shown by lithium is independent of GSK3*β*, as Figures 11(a) and 11(b) show that the GSK3*β* inhibitor, SB216763, has no inhibitory effects on ROS and RANTES production. This study correlates with other studies showing that lithium's influence on production of inflammatory mediators is independent of GSK3*β* [15, 20].

The process used by different receptors in mediating activation of the noncanonical NF-*κ*B signalling pathway remains unclear. The TNFR-associated factor (TRAF) proteins may play an important role, since they are known to be recruited to various TNFR family members. Interestingly, a common TRAF molecule recruited by the different noncanonical NF-*κ*B-stimulating receptors is TRAF3 [29]. TRAF3 is a negative regulator of NF-kappa-B-inducing kinase (NIK), a key signalling molecule involved in noncanonical NF-*κ*B activation. TRAF3 physically interacts with NIK and targets NIK for degradation by the proteasomes. Remarkably, the receptor-mediated activation of noncanonical NF-*κ*B signalling is associated with persistent degradation of TRAF3 and the marked elevation of NIK expression [14, 29]. Findings in the present study suggest that inhibition of NF-*κ*B signalling could involve upregulation of TRAF3 by lithium which would set up NIK for TRAF3-mediated destruction and result in inhibition of inflammation.

Inflammation triggered by LPS-TLR4 interaction culminates in activation of the NF-*κ*B pathway through dimerization of the p65 and p50 NF-*κ*B subunits. NF-kappa B is an important activator of immune and inflammatory response genes. NF-kappa B is sequestered in the cytoplasm of nonstimulated cells through interaction with the I*κ*B-*α* inhibitors. These inactive complexes dissociate in response to a variety of extracellular signals, thereby allowing free NF-kappa B dimers to translocate to the nucleus and activate transcription of specific target genes. Phosphorylation of I*κ*B-*α* is responsible for dissociation of the inactive complexes, an event that is rendered irreversible by rapid I*κ*B degradation. Inhibitor of kappa B-*α* is known to block the DNA-binding activity of NF-*κ*B p65-p50 using its DNA dissociation abilities embedded on its C-terminal [30]. In addition to I*κ*B-*α*, the p50 homodimer is documented to inhibit the transcriptional role of p50-p56 heterodimer since it possesses the DNA binding domain and not the transcriptional activation domain. It is, therefore, thought to block the kappa responsive site [31]. The observation made in this current study is that lithium upregulates p50 and I*κ*B-*α* mRNA expression (Figures 9 and 10) and inhibits translocation of NF-*κ*B from cytosol to the nucleus (Figure 8). Upregulation of these NF-*κ*B regulators could be responsible for lithium-mediated inhibition of inflammation or oxidative stress in these activated macrophages.

Translocation of NF-*κ*B to the nucleus leads to DNA-binding and transcription of several inflammatory target sites. One of these target sites in macrophages is inducible nitric oxide synthase (iNOS), which is responsible for NO production. Transcription of iNOS and oxidative stress genes has been reported to be involved in the pathogenesis of a number of diseases and disorders [28]. Oxidative stress results in oxidation of proteins, lipids, and nucleic acids that eventually evoke malfunctioning cells and cell demise, leading to diseases and disorders [7]. This study suggests that the anti-inflammatory properties of lithium in LPS-activated Raw 264.7 macrophage cells could be attributed to the observed inhibition of ROS and RNS production. In this study, the florigenic cell permeant, H_2_DCF-DA, is converted to a highly fluorescent DCF through removal of the acetate groups by intracellular esterases, leading to oxidation by ROS. Results from this assay suggest that the treatment of Raw 264.7 macrophages with lithium downregulates LPS-mediated ROS production. This is illustrated by the low fluorescence intensity observed in macrophage cells challenged with both lithium and LPS-activated cells in contrast to cells treated with LPS alone (Figure 5). This observation is in agreement with results observed in recent studies obtained from the treatment of glia cells and cancer cells with lithium [24, 32].

Lithium concentrations from 1.25 to 5 mM showed a significant downregulation of NO production in Raw 264.7 macrophage cells. Cells activated with LPS and treated with 1.25 to 5 mM lithium have shown low production of pink colour that embodies NO_2_^⁻^ as compared to LPS-activated cells during Griess assay (Figure 6)_._ Evaluation of NO production was further pursued using florigenic cell permeants and NO indicator, DAF2-DA, which fluoresces green upon interaction with NO. Lithium concentrations of up to 5 mM showed low DAF fluorescence intensity, which represents low production of NO (Figures 7(a) and 7(b)). Lower concentrations of lithium seem to have a profound inhibitory effect on NO production in LPS-activated macrophages; this could be as a result of the previous suggested narrow activity spectrum of lithium [2]. Many studies investigating the effect of lithium on NO production reported inconsistent findings dependent on cell type and nature of experimental setup. Several studies reported that lithium enhances NO production whereas others showed that lithium inhibits the NO pathways. Other studies reported no significant changes in NO production patterns of cells treated with lithium [14, 33, 34].

There seems to be more to lithium than its successful use in the management of bipolar disorders. Results from this study and other recent findings point towards the potential use of lithium in the management of neurodegenerative disorders [14]. Its neuroprotective property is suggested to emanate from suppression of proapoptotic proteins such as p53 and activation of antiapoptotic proteins such as Bcl-2, heat shock protein-40 and modulation of PI3K/Akt survival signalling pathway [2]. Lithium has played a significant role in psychiatry for more than 60 years as a gold standard mood stabilizer with desirable outcomes [1, 35]. In addition to its pivotal role in manic ailments, studied have shown its great involvement in inflammation. It has been shown to ameliorate multiple sclerosis through attenuation of proinflammatory cytokines, leukocyte infiltration through endothelial barrier, and inactivating the microglia cells activity that is known to be causative of neuronal cells demyelination [36]. Moreover, Wang and colleagues have recognised attenuation of type I interferon both *in vivo* and *in vitro* after challenging cells and mice with Sendai virus (seV). Interestingly, mice treated with lithium did not develop severe leukocyte extravasation or any tissue damage signs as recognised in lithium-untreated mice [20].

Although extensive research has been done towards the possible use of lithium as a potential anti-inflammatory agent, the exact mechanism of lithium remains unknown and molecular targets associated with its observed outcomes remains unclear. Most research about lithium focused on its effects in neuropsychiatric models or cells associated with the central nervous system (e.g., microglia, astrocytes, and oligodendrocytes) and various molecular targets in this area have been studied [14]. Limited research has focused on the effects of lithium in innate immunity by studying its effects on mammalian macrophages. It was, therefore, the intention of this study to provide more information on molecular targets of lithium by evaluating the effect of lithium on NF-*κ*B activity and mRNA expression levels of genes associated with regulation of oxidative stress and inflammation in a nonneuronal model of Raw 264.7 macrophages so as to further understand its actions beyond bipolar disorders. Interestingly, other studies have reported that lithium appears to preserve or increase the volume of brain structures involved in emotional regulation such as the prefrontal cortex, hippocampus, and amygdala, possibly reflecting its neuroprotective effects on neuronal and noncancerous mammalian cells [37].

Various cytotoxicity and apoptosis assays were used in this study to confirm that lithium does not induce cell death in the Raw 264.7 macrophage cells (Figures 1–4). The results are in agreement with previous findings showing the selective cytotoxic effect of lithium on cancerous cells while sparing their noncancerous counterparts [24, 38–40]. Moreover, this work supports the reliable safe use of lithium in clinical health and its hematopoietic role [41, 42]. Unlike other reports from studies that used neuronal cells and neuronal macrophages, our findings show the effects of lithium and LPS on proliferation of nonneuronal macrophages in real time while observing accompanying changes in oxidative stress levels. These cellular profiles of lithium which correlated with reduced oxidative stress also support research findings obtained using mammalian cells of neurological origin *in vitro* and *in vivo albeit* at varying concentrations [24, 43].

## 4. Conclusion

This study is the first to show that lithium inhibits the production of reactive nitrogen and oxygen species in activated Raw 264.7 macrophages without altering cell proliferation, growth, morphology, or viability in real time while modulating mRNA expression of Tollip, Traf-3, I*κ*B*α*, and NF-*κ*B1/p50. It should be noted that these effects were obtained mainly under the treatment with an extratherapeutic plasma concentration (less than 1.2 mM) of lithium permissible in patients taking the drug, suggesting that perhaps lithium might be more effective in combination therapy to permit lower dosages with synergistic drugs. This study suggests that the anti-inflammatory mode of action of lithium involves inhibitory proteins such as p50, Tollip, TRAF3, and I*κ*B-*α*. The ability of lithium to exert these anti-inflammatory effects without inducing cell death in macrophages makes it a promising candidate for the treatment of inflammation-based diseases such as cancer, Alzheimer's and Parkinson's diseases, and other neurodegenerative disorders. The affordability and extensive distribution of lithium will make this type of treatment accessible to a much wider population.

## 5. Materials and Methods

### 5.1. Cell Cultures

Raw 264.7 macrophage cells were obtained from Prof. Gordon Brown (Institute of Infectious Diseases and Molecular Medicines, University of Cape Town) (RSA). These cells were maintained in cell culture flasks at 37°C, in a humidified 95% air and 5% CO_2_ atmosphere. Determination of cell density was executed using trypan blue exclusion assay. This assay was accomplished by diluting cell 10x with trypan blue dye, and then the cells were counted using hemocytometer under the inverted light microscope.

### 5.2. xCELLigence Real-Time Cell Analyser

The real-time cell analyser system was used to evaluate cellular integrity and proliferative and cytotoxic effect of lithium on Raw 264.7 macrophage cells in real time. xCELLigence is a current-based cell sensor that relates electrical impedance to cell status/cell index as determined by cell morphology, cell adhesion, and cell number. This system was set and initiated according to the manufacturer's protocol. Cells were seeded in microelectrical cell sensor plates at a density of 40,000 cells/well for 24 hrs and then treated with 0.02 mg/ml actinomycin-D, 10 mM NaCl, and various concentrations of lithium chloride (1.25, 2.5, 5, 10, 20, 50, and 100 mM) for 56 hrs. The xCELLigence (RTCA) software was set to take readings after every 15 min interval (Roche, USA).

### 5.3. Cell Viability and Proliferation Assay

In order to assess proliferative and cytotoxic effects of lithium on Raw 264.7 cells, a colorimetric MTT assay that relies on the mitochondrial succinate dehydrogenase of viable cells was employed. This assay was initiated by seeding cells (Raw 264.7 macrophages) in a 96-culture-well plate at a density of 6 × 10^6^ cell/ml and incubated overnight. Thereafter, cells were treated with various concentration of LiCl (0.6125, 0.325, 1.25, 2.5, 5, 10, 20, 50, and 100 mM), 10 mM NaCl, and 0.02 mg/ml actinomycin-D (Sigma Aldrich, USA) for 24 hr. Cell imaging was done using a light microscope prior to the addition of 5 mg/ml of MTT (Fluka BioChemika, Switzerland) for 4 hr. After incubation, medium was aspirated and 100 *μ*l of DMSO (Saarchem, RSA) was added to each well and the plates were incubated for an hour. This was then followed by measuring absorbance at 570 nm using GloMax-Multi microplate reader (Promega, USA).

### 5.4. Annexin-V Fluorescein-5-isothiocyanate (FITC), Propidium Iodide (PI), and (4′,6-Diamidino-2-phenylindole) DAPI Apoptosis Detection Assay Using Fluorescent Microscopy

The effects of 10 mM lithium on the induction of apoptosis in Raw 264.7 cells were assessed using Annexin-V FITC/PI kit (BD Biosciences, USA) according to the manufacturer's protocol. This assay kit detects the flipping of plasma membrane inward out, membrane integrity, and DNA fragmentation. Cells were seeded at a density of 6 × 10^6^ cell/ml on coverslips in six-well plates and incubated overnight. Cells were treated with 10 mM NaCl, 10 mM LiCl, and 0.02 mg/ml actinomycin-D for 24 hr. This was followed by the removal of medium and washing of cells with 1x PBS. Cells were stained with PI and Annexin-V for 20 min in the dark at room temperature (RT). Cells on the coverslips were fixed for 30 min with 3.7% paraformaldehyde. Coverslips were then mounted with mounting medium on microscope slides and photographed using Nikon Ti-E inverted fluorescent microscope (Nikon, Japan).

### 5.5. Annexin-V FITC and (PI) Apoptosis Detection Assay Using Flow Cytometer

Flow cytometry was used to detect and quantify LPS-activated macrophage cells undergoing apoptosis after treatment with varying concentrations of lithium. In order to examine the apoptotic effects of lithium on macrophages, cells were seeded at a density of 6 × 10^6^ cell/ml in 60 mm cell culture dishes and incubated overnight. Cells were treated with 10 mM NaCl, 10 and 20 *μ*g/ml actinomycin-D, and 5, 10, 20, and 50 mM LiCl for 24 hr. This was followed by the removal of medium and washing of cells with 1x PBS. Cells were stained with PI and Annexin-V for 20 min in the dark at RT; this was then followed by washing and suspending cells in 1x PBS. Suspended cells were analysed using a flow cytometer according to the manufacturer's protocol (BD Biosciences, USA).

### 5.6. Griess Reagent Assay

Griess assay is a colorimetric technique that measures nitrite (NO_2_‐), one of the two primary stable forms of nitric oxide. This assay relies on the reaction described in 1879 by Griess [16]. In order to assess the effects of lithium on nitric oxide (NO) production, cells were seeded in 96-well plates at a density of 6 × 10^6^ cell/well in a phenol-free medium overnight. Cells were pretreated for 1 hour with various concentrations of lithium (5 and 10 mM) or 10 mM NaCl and activated with 10 *μ*g/ml LPS (Sigma, USA) for 24 hr. After 24 hr of treatment, nitric oxide was measured by mixing equal amounts of phenol-free medium and Griess reagent (Sigma-Aldrich, USA). The absorbance was measured using GloMax-Multi microplate reader at 550 nm (Promega, USA).

### 5.7. DAF2-DA Nitric Oxide Measurement Assay

The DAF2-DA assay is a fluorescence-based NO detection assay that uses a cell permanent 4,5-diaminofluorescein diacetate (DAF2-DA) as NO detector (Sigma-Aldrich, USA). Diacetate (DA) part of DAF2-DA is hydrolysed by cellular esterases to produce DAF2 compound that reacts with NO to produce a fluorescent triazolofluorescein (DAF-2T). Therefore, in this study, cells were seeded on coverslips in 6-well plates overnight and then pretreated with LiCl (5 and 10 mM), 10 mM NaCl and activated with 100 ng/ml Ultrapure LPS (Invitrogen, USA) for 24 hrs. Thereafter, cells were stained with 10 *μ*m/ml DAF2-DA for 20 min at RT and washed with 1xPBS. Cells were further stained with 25 *μ*g/ml Hoechst for 20 min at RT, thereafter followed by fixing with 2.7% paraformaldehyde for 30 min. Nitric oxide quantitation was accomplished using a Nikon Ti-E inverted fluorescent microscope (Nikon, Japan).

### 5.8. Oxidative Burst Assay

In an attempt to analyse the effects of lithium on oxidative burst, a Phagoburst assay kit (Glycotope, Germany) was employed and the oxidative burst assays were performed according to the manufacturer's protocol. This is a fluorescence-based assay that uses a florigenic cell permeant, H_2_DCF-DA, that is converted to a highly fluorescent DCF through the removal of the acetate groups by intracellular esterases and subsequent oxidation. In summary, cells were seeded at a density of 6 × 10^6^ cell/ml on coverslips in 6-well plates overnight. Adherent cells were then pretreated with 10 mM LiCl and activated with 10 *μ*g/ml LPS for 24 hr. Thereafter, the cells were washed once with a wash buffer and 50 *μ*l of H_2_DCFDA was added in all plates followed by a 30 min incubation in the dark at RT. Cells were then washed with a washing solution to remove excess dye and 50 *μ*l of DNA staining solution was added. Cells were incubated with DNA staining solution for 20 min at RT in the dark. DNA staining solution was then removed, and 3.7% paraformaldehyde was added for 30 min to fix the cells. This was followed by mounting of coverslips to the microscope and examination. On the second H_2_DCF-DA assay, the assay was the same as the first one with the addition of 10 *μ*M SB216763, which was used to treat the cells and then stimulated with 5 mg/ml LPS. Then, fluorescence intensity was measured at 20x magnification with EVOS FL Colour imaging system (Life Technologies, USA).

### 5.9. Immunocytochemistry

Translocation properties of NF-*κ*B will be assessed by immunofluorescence staining technique. Cells were cultured at a density of 6 × 10^5^ cells/ml on coverslips and allowed to adhere for 4 hrs. Thereafter, cells were treated with 10 mM LiCl, 10 mM NaCl, 100 ng/ml ultrapure LPS, and a combination of salts and LPS for 90 min. Cells were then washed with 1x PBS and fixed at RT for 30 min with 3.7% paraformaldehyde. Thereafter, cells were washed with 1x PBS and permeabilised with a permeabilising buffer (0.1% Triton X-100, 1% BSA in 1x PBS) for 30 min. The nonspecific binding was blocked with 1% BSA in 1x PBS for an hour. Thereafter, cells were incubated with anti-NF-kB antibody (1 : 500) for an hour, and then the cells were washed 3x with 1x PBS and stained for 30 min with anti-rabbit IgG-fluorescence (1 : 2000). After 30 min incubation, cells were washed with 1x PBS and then followed by DNA staining with 25 *μ*g/ml Hoechst for 20 min at RT. Thereafter, coverslips were mounted on glass slides with mounting medium and then NF-*κ*B translocation was analysed under fluorescence microscope (Nikon Eclipse TS100F, Japan).

### 5.10. Elisa Assay RANTES Production Determination

In order to measure the production of RANTES/CCL-5, Raw 264.7 cells were seeded at 1 × 10^6^ Cells for 24 hrs in T25 flasks and then cells were treated with 10 mM LiCl, NaCl, and 10 *μ*M SB216763, thereafter activated with 5 mg/ml LPS for 24 hrs. The supernatant was collected after 24 hrs, and then enzyme-linked immunosorbent assay (ELISA) was executed according to the manufacturer's protocol (PeproTech, USA). The OD was measured at 405 nm with ELx 802 universal microplate reader (BioTek Instruments, Inc.).

### 5.11. Real-Time PCR Analysis

Raw 264.7 cells were cultured in a 60 mm cell culture dishes overnight at a density of 6 × 10^6^ cell/ml. Thereafter, cells were treated with 10 mM LiCl, 10 *μ*m/ml LPS, and a combination of 10 mM LiCl and 10 *μ*m/ml LPS for 24 hrs. Total RNA was isolated using RNeasy Mini Kit according to the manufacturer's procedure (Qiagen, USA) and then cDNA was synthesised as specified by RT^2^ first-strand kit manufacturer's protocol (Qiagen, USA). In order to analyse gene expression in each sample, a 96-well RT^2^ profiling PCR Array system was used; accession numbers of target genes are specified in Table 1. A reaction mixture composed of 1350 *μ*l mixture of 2x RT^2^ SYBR Green Master Mix, 102 *μ*l cDNA synthesis reaction, and RNase-free water was formulated. Prior to the addition of PCR components mixture, the array plates were briefly centrifuged at low speed. Twenty-five microliters of PCR components mixture was added to each well of RT^2^ profiling PCR arrays coated with different target primers and the RT^2^ profiling PCR arrays were tightly sealed with optical thin-wall 8 strips. Before cycling, the arrays were briefly centrifuged at 1000 ×g for 1 min at 25°C. Thereafter, 40 cycles were prepared; 1 cycle for 10 min at 95°C, 40 cycles of 15 s at 95°C, and 1 min cycle at 60°C.

## Figures and Tables

**Table 1:** NF*κ*B pathway gene table with accession numbers of the primer genes used in the profiler real-time PCR array assay in LPS-activated Raw 264.7 macrophages.

NF-*κ*B pathway associated gene	Description	UniGene	GeneBank
Tollip	Toll interacting protein	Mm.103551	NM_023764
Traf3	TNF receptor-associated factor 3	Mm.27431	NM_011632
NF-*κ*Bia/I*κ*B-*α*	Nuclear factor of kappa light polypeptide gene enhancer in B-cells inhibitor, alpha	Mm.170515	NM_010907
NF-*κ*B1/p50	Nuclear factor of kappa light polypeptide gene enhancer in B-cells 1, p105	Mm.256765	NM_008689

## Data Availability

The raw data are available from the corresponding author upon reasonable request.

## Abbreviations

FDA: Food and Drug Administration
MØ: Macrophages
LPS: Lipopolysaccharide
H2DCF-DA: 2′, 7′-Dichlorodihydrofluorescein diacetate
RHD: Rel homology domain
PSN: Penicillin, streptomycin, and neomycin
FBS: Fetal bovine serum
DAF-2DA: 5, 6-Diaminofluorescein diacetate
ROS: Reactive oxygen species
NO: Nitric oxide
NO_2_^−^: Nitrite
MTT: 3-(4, 5-Dimethylthiazol-2-yl)-2, 5-diphenyltetrazolium bromide
GSK-3: Glycogen synthase kinase-3
NF-*κ*B: Nuclear factor-κB
I*κ*B-*α*: Inhibitor of kappa-B kinase alpha
TRAF3: TNF receptor-associated factor 3
Tollip: Toll interacting protein
Tcf-Lef: T-cell factor-lymphoid enhancing factor
*γ*-GCS: *γ*-Glutamylcysteine synthetase
PI3K: Phosphoinositol 3-kinase
p53: Tumor suppressor.
